# The medical food Souvenaid affects brain phospholipid metabolism in mild Alzheimer’s disease: results from a randomized controlled trial

**DOI:** 10.1186/s13195-017-0286-2

**Published:** 2017-07-26

**Authors:** Anne Rijpma, Marinette van der Graaf, Marieke M. Lansbergen, Olga Meulenbroek, Aysun Cetinyurek-Yavuz, John W. Sijben, Arend Heerschap, Marcel G. M. Olde Rikkert

**Affiliations:** 1 Department of Geriatric Medicine, Radboud University Medical Center, P.O. Box 9101, 6500 HB Nijmegen, The Netherlands; 2 Radboudumc Alzheimer Center, Donders Institute for Brain, Cognition and Behavior, Radboud University Medical Center, Nijmegen, The Netherlands; 3 Department of Radiology and Nuclear Medicine, Radboud University Medical Center, Nijmegen, The Netherlands; 4 Department of Pediatrics, Radboud University Medical Center, Nijmegen, The Netherlands; 5 Nutricia Research, Nutricia Advanced Medical Nutrition, Utrecht, The Netherlands

**Keywords:** Nutrition, Phospholipid metabolism, Magnetic resonance spectroscopy, ^31^P-MRS, ^1^H-MRS, Medical food, Alzheimer’s disease, Dementia, Souvenaid, Fortasyn Connect

## Abstract

**Background:**

Synaptic dysfunction contributes to cognitive impairment in Alzheimer’s disease and may be countered by increased intake of nutrients that target brain phospholipid metabolism. In this study, we explored whether the medical food Souvenaid affects brain phospholipid metabolism in patients with Alzheimer’s disease.

**Methods:**

Thirty-four drug-naive patients with mild Alzheimer’s disease (Mini Mental State Examination score ≥20) were enrolled in this exploratory, double-blind, randomized controlled study. Before and after 4-week intervention with Souvenaid or an isocaloric control product, phosphorus and proton magnetic resonance spectroscopy (MRS) was performed to assess surrogate measures of phospholipid synthesis and breakdown (phosphomonoesters [PME] and phosphodiesters [PDEs]), neural integrity (*N*-acetyl aspartate), gliosis (*myo*-inositol), and choline metabolism (choline-containing compounds [tCho]). The main outcome parameters were PME and PDE signal intensities and the PME/PDE ratio.

**Results:**

MRS data from 33 patients (60–86 years old; 42% males; Souvenaid arm *n* = 16; control arm *n* = 17) were analyzed. PME/PDE and tCho were higher after 4 weeks of Souvenaid compared with control (PME/PDE least squares [LS] mean difference [95% CI] 0.18 [0.06–0.30], *p* = 0.005; tCho LS mean difference [95% CI] 0.01 [0.00–0.02], *p* = 0.019). No significant differences were observed in the other MRS outcome parameters.

**Conclusions:**

MRS reveals that Souvenaid affects brain phospholipid metabolism in mild Alzheimer’s disease, in line with findings in preclinical studies.

**Trial registration:**

Netherlands Trial Register, NTR3346. Registered on 13 March 2012.

**Electronic supplementary material:**

The online version of this article (doi:10.1186/s13195-017-0286-2) contains supplementary material, which is available to authorized users.

## Background

Synaptic dysfunction is a major contributing factor to cognitive impairment in Alzheimer’s disease (AD) [1, 2] and may be caused by deficits in neuronal membrane composition and function [3, 4]. Because the neuronal membrane is composed mainly of phospholipids [5], interventions that target brain phospholipid metabolism may affect cognitive function in AD.

The most abundant phospholipids in the neuronal membrane are phosphatidylethanolamine (PE) and phosphatidylcholine (PC) [4, 5]. They are formed in the Kennedy cycle, wherein phosphomonoesters (PMEs) are converted to phospholipids that can then be incorporated into neuronal membranes [6]. The breakdown of these phospholipids releases phosphodiesters (PDEs), which can either be used for resynthesis or broken down further [7]. The synthesis of brain phospholipids is influenced by the availability of specific nutrients to the brain, consisting of rate-limiting phospholipid precursors [8, 9]. This is affected not only by nutritional intake but also by the intake of cofactors that influence precursor uptake and metabolism. Increasing the availability of several precursors proved to have a synergistic effect on phospholipid formation as well as on dendritic spine density [9–12]. Furthermore, circulating levels of most of these nutrients (precursors and cofactors) as well as brain choline levels are lower in patients with AD [13–15], thus lowering precursor availability. The specific multinutrient combination Fortasyn® Connect (FC) in the medical food Souvenaid® (Nutricia Advanced Medical Nutrition, Utrecht, The Netherlands) contains precursors and cofactors in the phospholipid synthesis pathway (docosahexaenoic acid [DHA]; eicosapentaenoic acid [EPA]; uridine monophosphate [UMP]; choline; phospholipids; selenium; folic acid; and vitamins B_6_, B_12_, C, and E), and it has been formulated to promote neuronal membrane (phospholipid) formation and function in AD [8]. Previous studies in animal models of AD and aging have shown that long-term supplementation with FC positively affects exploratory behavior and memory [16, 17], in addition to enhancing phospholipid synthesis and improving cholinergic transmission [18]. This indicates that supporting synaptic function by increasing phospholipid formation is a promising strategy to improve cognition or reduce cognitive decline.

Randomized controlled trials with patients with AD demonstrated improvement in memory performance in those with mild AD over 12–24 weeks of intervention with this specific multinutrient combination [19, 20] as well as altered functional connectivity and preserved brain network organization [20, 21]. However, the physiological underpinnings in humans remain to be further elucidated. The present study was designed to provide more insight into the hypothesized underlying mechanisms of FC (i.e., promoting neuronal membrane [phospholipid] formation and function) in humans.

Neither synapse number nor phospholipid membrane composition can be assessed directly in vivo, but phosphorus magnetic resonance spectroscopy (^31^P-MRS) allows for the noninvasive investigation of phospholipid building blocks and breakdown products (i.e., PME and PDE, respectively) [22]. In addition, using proton (^1^H) MRS, brain metabolites related to neural integrity (*N*-acetylaspartate [NAA]), gliosis (*myo*-inositol [mI]), choline metabolism (total choline [tCho]), and energy metabolism (total creatine [tCr]) can be assessed as well [22, 23]. Hence, in the present study, we explored whether the medical food Souvenaid affects brain phospholipid metabolism and neural integrity in patients with mild AD using ^31^P-MRS and ^1^H-MRS.

## Methods

### Subjects and design

The MRS AD study was a 4-week, randomized, controlled, double-blind, single-center trial with patients with mild AD. Significant effects of FC on memory performance were reported in this population previously [19, 20]. In the present study, we explored whether FC affects brain phospholipid metabolism in patients with mild AD. All visits took place between October 2012 and February 2015 at the Radboud University Medical Center (Nijmegen, The Netherlands). AD drug-naive patients aged ≥50 years with a diagnosis of probable or possible AD (according to the revised 2011 criteria of the National Institute of Neurological and Communicative Disorders and Stroke and Alzheimer’s Disease and Related Disorders Association [24]) with evidence of the pathophysiological process (i.e., from structural magnetic resonance imaging [MRI] or cerebrospinal fluid [CSF] biomarker assays), a Mini Mental State Examination (MMSE) score ≥20, and a Geriatric Depression Scale (GDS) score ≤6 (of 15) were recruited from the hospital’s memory clinic or by referral from regional hospitals. All subjects were drug-naive for AD medication (cholinesterase inhibitors and *N*-methyl-d-aspartate antagonists) and were free of neurological or psychiatric disorders (other than dementia). To increase subject recruitment, eligibility criteria were extended during the study to include patients who were off AD medication for at least 3 months prior to the study, but no additional subjects were recruited who did not meet the original criteria. Subjects did not consume oily fish more than twice per week or use nutritional supplements containing DHA, EPA within 2 months prior to baseline, or nutritional supplements containing >200% of the recommended daily allowance of vitamins B_6_ (2.8 mg), B_12_ (5 μg), C (160 mg), E (24 mg), or folic acid (0.4 mg) within 1 month prior to baseline. Use of anticholinergic or antipsychotic medication and of other medical foods or investigational products was also prohibited within 1 month prior to baseline, as well as changes in dose of lipid-lowering medication, antihypertensives, and/or antidepressants. All subjects were screened for MRI contraindications before inclusion in the study. Written informed consent was obtained from all patients and their informal caregivers. The local ethics committee reviewed and approved the protocol. The study was conducted in accordance with the Declaration of Helsinki and is registered in the Netherlands Trial Register (NTR3346).

Medical history, medication and nutritional supplement use, MMSE score, GDS score, date of birth, sex, ethnicity, smoking habits, alcohol consumption, and family history of AD were recorded for all subjects at the first (screening) visit. Eligible subjects were randomly allocated to receive either the test product (i.e., Souvenaid containing the specific nutrient combination FC) or an isocaloric control product once daily as a drink for a double-blind period of 4 weeks (1:1 randomization, stratified on the basis of sex). The test product contained DHA; EPA; phospholipids; choline; UMP; selenium; folic acid; and vitamins B_6_, B_12_, C, and E (Table 1). On the basis of previous publications in which ^31^P-MRS brain metabolites in humans were already affected after 1-week administration of uridine [25] and 6-week administration of citicoline [26], it was expected that an intervention period of 4 weeks was sufficient to observe an effect of the test product on ^31^P-MRS brain metabolites.

**Table 1:** Nutritional composition of the study products (amount per daily dose of 125 ml)

	Control	Active
Energy	125 kcal	125 kcal
Protein	3.8 g	3.8 g
Carbohydrate	16.5 g	16.5 g
Fat	4.9 g	4.9 g
EPA	0	300 mg
DHA	0	1200 mg
Phospholipids	0	106 mg
Choline	0	400 mg
UMP	0	625 mg
Vitamin E (α-tocopherol equivalents)	0	40 mg
Vitamin C	0	80 mg
Selenium	0	60 μg
Vitamin B_12_	0	3 μg
Vitamin B_6_	0	1 mg
Folic acid	0	400 μg

*EPA* Eicosapentaenoic acid, *DHA* Docosahexaenoic acid, *UMP* Uridine monophosphate

Test product is Souvenaid, containing the specific nutrient combination Fortasyn Connect. Souvenaid and Fortasyn are registered trademarks of Nutricia N.V

Numbered and sealed randomization envelops (containing one of four codes, two representing the test group and two the control group) were generated at Nutricia Research by the clinical studies supplies manager and opened upon randomization on-site by the investigator. All subjects and any person involved in subject recruitment, group allocation, data acquisition and processing, or statistical analyses were blinded to the intervention group (test or control) until the analyses of the main outcome parameters were completed. The study product was packaged in tetra-packs (until 15 January 2014) or plastic bottles (from 15 January 2014 onward) and labeled with one of the four randomization codes. With assistance from their informal caregivers, subjects recorded intake of the product daily in a diary, which was used to verify intake at the week 4 visit.

At baseline and after 4 weeks, venous blood samples were taken and magnetic resonance (MR) measurements were performed. MR measurements at week 4 took place at least 2 h after intake of the last study product. For the purposes of compliance and safety, a phone call was conducted after 14 days of product intake. A final follow-up call was conducted 2 weeks after the last visit. Subjects were instructed to minimize intake of high-choline food on the days of the baseline and week 4 visits and to keep intake of concomitant nutritional supplements and medication stable (unless deemed necessary by their physician) during the study. Analyses of the blood samples were performed as reported previously [27].

### MR protocol

MRI and MRS were performed on a MAGNETOM Trio Tim System 3-T MR system (Siemens Healthcare, Erlangen, Germany) with a dual-tuned ^1^H/^31^P volume head coil (Rapid Biomedical, Würzburg, Germany). High-resolution structural MR images were acquired with a T1-weighted magnetization-prepared rapid gradient echo sequence (repetition time [TR] 2300 milliseconds, echo time [TE] 3.16 milliseconds, inversion time 1100 milliseconds, 15-degree flip angle, 176 sagittal slices, slice matrix size 256 × 256, slice thickness 1 mm, voxel size 1 × 1 × 1 mm, acquisition time [TA] 6:25 minutes).^31^P-MR spectra were acquired by whole-brain 3D MR spectroscopic imaging (MRSI; TR 2000 milliseconds, 40-degree flip angle, four averages, acquisition delay 0.10 milliseconds, broadband proton decoupling applied during first half of 512-millisecond acquisition duration, field of view (FOV) 260 × 260 × 260 mm; matrix size 10 × 10 × 10, TA 13:08 minutes). *k*-Space was sampled with a weighted elliptical phase-encoding scheme with four averages. The FOV was centered on the midline and parallel to the line from the anterior commissure to the posterior commissure. Spatial postprocessing consisted of zero-filling to a matrix size of 16 × 16 × 16 followed by spatial Fourier transformation. The nominal volume of the selected cubic voxels is about 17.5 cm^3^; because of the spatial response function, the spectra have contributions from a spherical area with an effective volume of about 40 cm^3^ at 64% of this function [28].

^1^H-MR spectra of the hippocampus were acquired with a single-slice 2D semilocalization by adiabatic selective refocusing (sLASER) MRSI sequence, with water suppression enhanced through T1 effects (TR 2100 milliseconds, TE = 30 milliseconds, 90-degree excitation flip angle, six averages, FOV 120 × 160 × 10 mm, nominal voxel size 3.75 × 5 × 10 mm), positioned parallel to the hippocampi. ^1^H-MR spectra of the anterior and posterior cingulate cortices (ACC and PCC, respectively) were acquired with a single-voxel point resolved spectroscopy (PRESS) sequence with chemical shift selective water suppression (TR 3000 milliseconds, TE 30 milliseconds, 90-degree excitation flip angle, 64 averages, voxel size 27 × 15 × 15 mm [ACC] and 20 × 20 × 16 mm [PCC]). With each sequence, an additional MR spectrum without water suppression was obtained (2D sLASER: one average, PRESS: eight averages). The complete MR protocol, including positioning of the patient, took approximately 90 minutes. Because the ^31^P-MRS quantities PME, PDE, and PME/PDE were the main outcome measures, ^31^P-MRS measurements were always performed before ^1^H-MRS measurements in case subjects failed to complete the entire scanning protocol.

### MR data analysis

**Fig. 1:**
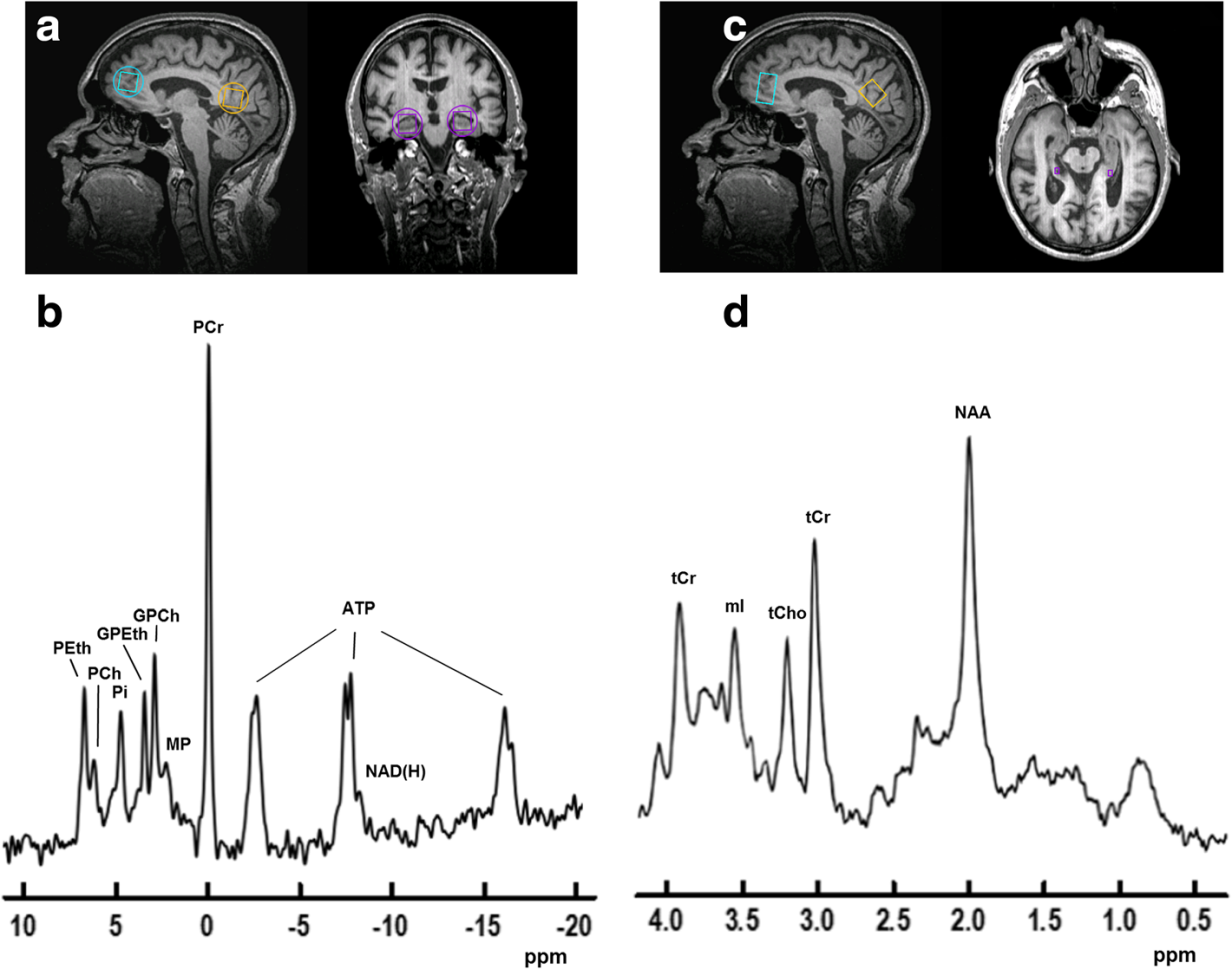
Voxel selection and representative examples of ^31^P and ^1^H magnetic resonance (MR) spectra. **a** Voxel selection of ^31^P-MR spectra displayed on sagittal (*left*) and coronal (*right*) anatomical images: anterior cingulate cortex (*blue*), retrosplenial cortex (*yellow*), left and right hippocampus (*purple*). The nominal voxel size is indicated by *squares*, and the approximation of the effective spherical voxel size is indicated by *circles*. **b** Representative ^31^P-MR spectrum from the retrosplenial cortex. Zero-filling to 4096 data points and an 8-Hz Gaussian filter were applied. **c** Sagittal (*left*) and transversal (*right*) anatomical images showing single-voxel volumes of ^1^H-MR spectra in anterior cingulate cortex (*blue*), posterior cingulate cortex (*yellow*), and voxel selection of left and right hippocampi (*purple*). **d** Representative ^1^H-MR spectrum from the posterior cingulate cortex. Zero-filling to 8192 data points and a 2-Hz Lorentzian filter were applied. PEth, Phosphoethanolamine; PCh, Phosphocholine; Pi, Inorganic phosphate; GPEth, Glycerophosphoethanolamine; GPCh, Glycerophosphocholine; MP, Membrane phospholipids; PCr, Phosphocreatine; ATP, Adenosine triphosphate; NAD(H), Nicotinamide adenine dinucleotide; ppm, Parts per million; NAA, *N*-acetyl-aspartate; tCho, Choline-containing compounds; tCr, Total creatine; mI, *Myo*-inositol

Four regions of interest (ROIs) were selected for analysis from the 3D ^31^P-MRSI data: ACC, retrosplenial cortex (RSC), and left and right hippocampi (HL and HR, respectively). Voxels covering these ROIs were selected by shifting the MRSI grid in the *x*, *y*, and *z* dimensions using anatomical landmarks on the T1-weighted anatomical images. From the 2D ^1^H-MRSI data, two voxels positioned on the hippocampi (HL and HR, respectively) were selected for analysis, in addition to the single-voxel data from the ACC and PCC (Fig. 1a, c).

The Metabolite Report software package (Siemens Healthcare) was used for postprocessing (i.e., zero-filling, phase correction, 100-millisecond exponential filter, baseline correction) and for automatic fitting of the ^31^P-MR spectra in the time domain using prior knowledge (unpublished data Rijpma and de Graaf [29]). Eleven well-resolved resonance peaks were fitted: the phospholipid metabolites phosphoethanolamine (PEth), phosphocholine (PCh), glycerophosphoethanolamine (GPEth), and glycerophosphocholine (GPCh); the high-energy phosphorus molecules phosphocreatine (PCr); adenosine triphosphates (ATPs) α-ATP, β-ATP, and γ-ATP; nicotinamide adenine dinucleotide [NAD(H)] and inorganic phosphate (Pi); and membrane-bound phospholipid (MP). The intensity of each metabolite resonance was expressed as a percentage area of the total phosphorus signal in the spectrum. Thus, the data are corrected for CSF content and for differences in atrophy. PME was calculated as the sum of PEth and PCh, PDE as the sum of GPEth and GPCh, and total adenosine triphosphate as the sum of α-ATP, β-ATP, and γ-ATP. In addition, the ratios PME/PDE, PEth/GPEth, PCh/GPCh, and PCr/Pi were computed. Intracellular pH was determined from the chemical shift difference between the PCr and Pi resonance peaks [30].

Both a quantitative evaluation of the fitting results and a visual quality control were performed. Quantitatively, only fits of metabolite peaks with a Cramér-Rao lower bound (CRLB) ≤30% were considered reliable. Qualitatively, two spectroscopists (AR and MvdG) independently checked the spectra by visual inspection of the original spectra and the fitting results. If a metabolite peak was visually present and its fit was assigned to the correct resonance, giving a minimal residue, the fitting result was accepted.

The LCModel software package (version 6.3-0C [31]) was used for postprocessing (e.g., eddy current correction and water scaling) and for automatic fitting of all ^1^H-MR spectra (i.e., two hippocampal voxels, ACC and PCC). The signal intensities of NAA, mI and glycine, and choline-containing compounds (tCho: PCh, GPCh, and free choline) were expressed relative to the intensity of creatine and phosphocreatine (tCr). Additionally, water-referenced levels of NAA, mI, tCho, and tCr, expressed in millimolar tissue concentration, and the ratio of NAA to mI were calculated. In the quantitative evaluation of the fitting results, a whole spectrum was rejected when the full-width half-maximum was >0.15 ppm (18.5 Hz) or the signal-to-noise ratio was <5, and only individual metabolite fits with a CRLB <30% were considered reliable. Qualitatively, two spectroscopists (AR and MvdG) independently judged the original spectra and the fitting results by visual inspection according to a prespecified set of criteria.

T1-weighted MR images from one visit were segmented into gray matter (GM), white matter (WM), and CSF using automatic segmentation software (SPM8, Welcome Trust Centre for Neuroimaging, London, UK; VBM8, Structural Brain Mapping Group, Jena, Germany). For each subject, the whole-brain GM fraction (GM/[GM + WM + CSF]) was calculated and used as a prespecified confounder in supportive covariate analyses.

### Statistical analyses

All statistical analyses were performed for the modified intention-to-treat (ITT) dataset, including all subjects who received at least one unit of the study product and had at least one MR measurement. The main statistical analyses of the MRS outcome parameters were also performed for the per-protocol (PP) population, which includes all subjects who had no major protocol deviations. All blood outcome parameters at week 4 were analyzed using analysis of covariance (ANCOVA) with between-subjects factors intervention group and sex, and with baseline measure as covariate.

All MRS outcome parameters at week 4 were analyzed using a predefined multilevel model with intervention group (test or control), sex, and brain region and its interaction with intervention group as fixed factors, considering brain region as a within-subject factor and adjusting for baseline. An unstructured variance-covariance matrix for brain region was selected. If the *p* value for intervention group by brain region interaction was <0.10, ANCOVA models per brain region were used. If the *p* value for the intervention by brain region interaction was >0.10, the interaction term was dropped from the model. For all fitted models, the influence diagnostics were used to explore the influence of different observations on the models. Analyses of the main ^31^P-MRS (PME, PDE, PME/PDE) and main ^1^H-MRS outcomes (NAA/tCr, mI/tCr, Cho/tCr, NAA/mI) were performed on partially unblinded data (using intervention group coded as X and Y), allowing analysis of the intervention effect while keeping the statisticians blinded to group allocation. The primary statistical analyses of these main outcome parameters were repeated with adjustments for possible confounders (i.e., MMSE, age, education level [low, medium, high], GM fraction, and intake of choline-containing food [only for choline-related outcome parameters]).

Additionally, the following predefined supportive analyses were performed on the main ^31^P-MRS (PME, PDE, PME/PDE) and main ^1^H-MRS (NAA/tCr, mI/tCr, Cho/tCr, NAA/mI) outcome parameters. First, the primary analyses described above were repeated using imputed baseline values when baseline values were missing. Thus, MRS outcome parameters at week 4 were analyzed using a predefined multilevel model with intervention group (test or control), sex, and brain region and its interaction with intervention group as fixed factors, considering brain region as a within-subject factor and adjusting for baseline, where missing baseline values were replaced by imputed baseline values (so-called multilevel model with imputation). Imputation of the baseline value was performed by a regression imputation using age, sex, and brain region. Second, linear mixed model analyses were conducted including the baseline value and the week 4 value of the outcome parameter in the outcome vector and including intervention group, sex, time, brain region, and two-way and three-way interactions between intervention group, brain region, and time as fixed factors, and a random intercept for time per subject. In this supportive model (so-called mixed model with time), the intervention effect is expressed by the interaction of time by intervention group. Third, the primary analyses were repeated for the one brain region for which most data were available. For all fitted models, the influence diagnostics were used to explore the influence of different observations on the models.

Statistical analyses were performed by both AR and ACY using SAS 9.2 and SAS 9.4 software, respectively (SAS Institute Inc., Cary, NC, USA). Statistical significance for the intervention effect was set at *p* < 0.05 without correction for multiple testing.

## Results

### Subjects

**Fig. 2:**
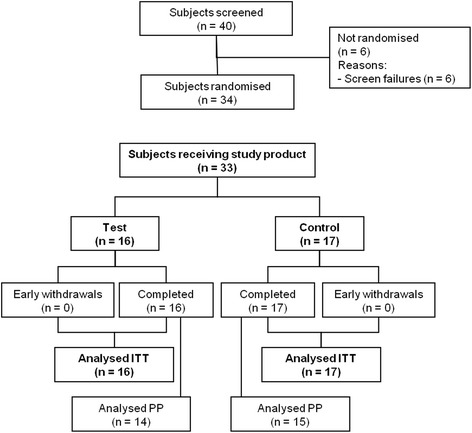
Flowchart of subjects in study. ITT, Intention to treat; PP, Per protocol

Of the 40 subjects who were screened, 34 subjects were included and randomized to receive either the test or the control product. One subject dropped out of the study without MR measurements and prior to product dispensing, owing to unexpected claustrophobia, which was an exclusion criterion for the present study as described in the study protocol. Hence, 33 subjects were included in the modified ITT population. Major protocol deviations were present for four subjects: no MR measurements at week 4 (*n* = 2) or (suspected) double product intake prior to week 4 measurements (*n* = 2). Hence, 29 subjects were included in the PP population (for a flowchart, *see* Fig. 2). Subjects in the test (*n* = 16) and control (*n* = 17) groups (modified ITT population) were comparable with respect to baseline characteristics (Table 2). The test group reported 80 medical conditions in the medical history, as compared with 120 medical conditions in the control group. Adherence to the study product, according to diary entries, was high (>96%) and equal in both groups.

**Table 2:** Subject characteristics

	Test (*n* = 16)	Control (*n* = 17)
Age, years	74.7 (4.8)	72.7 (8.2)
Male sex, *n* (%)	7 (44%)	7 (41%)
MMSE score	23.0 (2.1)	23.2 (1.8)
Time since diagnosis, months	1.7 [0.4-15.3]	1.4 [0.2-10.2]
BMI, kg/m^2^	24.2 (3.8)	26.7 (3.3)
Educational level, *n* (%)		
Low	6 (37.5%)	4 (23.5%)
Medium	8 (50.0%)	9 (52.9%)
High	2 (12.5%)	4 (23.5%)
Study product compliance	96.4% (4.8)	99.0% (1.6)

*AD* Alzheimer’s disease, *BMI* body mass index, *MMSE* Mini Mental State Examination

Education level: low, primary education or lower; middle, junior vocational training; high, senior vocational or academic training

Data are presented as mean (SD) or median [range] unless otherwise indicated

### Safety analysis and concomitant medication

In total, 27 adverse events (AEs) were reported, none of which were serious AEs. The number of subjects with at least one AE did not differ statistically between groups (test: 13 AEs in 8 subjects, control: 14 AEs in 7 subjects, *p* = 0.732 by Fisher’s exact test). A total of 15 of 33 subjects (45.5% of the total study population) reported at least one AE. AEs that were most often reported for both study groups concerned gastrointestinal system disorders (i.e., abdominal pain, diarrhea, dyspepsia, nausea, vomiting). The majority of AEs were considered to be unrelated to the study product. Seven AEs (five in the test group and two in the control group) had a relationship to the study product (“possibly” or “probably”), and were all of gastrointestinal nature (i.e., diarrhea, dyspepsia, nausea, vomiting). Regarding the use of concomitant medication (other than AD medication), 62.5% of subjects in the test group and 82.4% of the control group used concomitant medication until the 4-week intervention period. After the intervention period, 4 of 16 subjects in the test group and 8 of 17 subjects in the control group started AD medication (galantamine, memantine, or rivastigmine).

### Nutritional blood markers

Levels of uridine, choline, and vitamin E in plasma, as well as percentages of DHA, EPA, and total long-chain polyunsaturated fatty acids in plasma and in total fatty acids in erythrocyte membrane, were higher at week 4 in the test group than in the control group (all *p* < 0.001). Homocysteine levels were lower after 4 weeks of test product compared with the control product (*p* = 0.006). Docosapentaenoic acid in plasma or erythrocyte membrane was not significantly different at week 4 between the groups (*p* = 0.628 and *p* = 0.840, respectively). For details, *see* Table 3.

**Table 3:** Intervention effect on nutritional blood markers and homocysteine

Blood outcome parameter	Mean estimated value at week 4		Difference between groups at week 4	*p* Value for intervention effect
	Test (*n* = 16)	Control (*n* = 17)		
Fatty acids in the erythrocyte membrane				
DHA, %	4.9	2.8	2.1 (1.84 to 2.30)	<0.001
EPA, %	1.7	0.8	0.8 (0.69 to 0.98)	<0.001
DPA, %	1.6	1.6	0.014 (−0.12 to 0.15)	0.840
LC-PUFA (DHA + EPA + DPA), %	8.2	5.3	2.9 (2.51 to 3.24)	<0.001
Fatty acids in blood plasma				
DHA, %	4.4	1.5	2.9 (2.59 to 3.12)	<0.001
EPA, %	2.1	0.8	1.3 (1.10 to 1.51)	<0.001
DPA, %	0.50	0.49	0.008 (−0.03 to 0.04)	0.628
LC-PUFA (DHA + EPA + DPA), %	7.0	2.8	4.2 (3.79 to 4.53)	<0.001
Choline levels in blood plasma, μM	14.4	9.0	5.4 (3.39 to 7.47)	<0.001
Vitamin E levels in blood plasma, μM	50.9	39.6	11.4 (6.77 to 15.96)	<0.001
Homocysteine levels in blood plasma, μM	9.8	12.4	−2.6 (−4.31 to -0.82)	0.006
Uridine levels in blood plasma, μM	14.8	2.6	12.2 (7.24 to 17.11)	<0.001

*Abbreviations*: *DHA* Docosahexaenoic acid, *EPA* Eicosapentaenoic acid, *DPA* Docosapentaenoic acid, *LC-PUFA* Long-chain polyunsaturated fatty acids

The 95% CI is presented in parentheses

### MRS outcomes

Good-quality ^31^P-MR and ^1^H-MR spectra were obtained from all ROIs (Fig. 1b and d). If no significant interactions between intervention group and brain region were observed in the statistical analyses, only main intervention group effects are reported. Furthermore, the results from the covariate analyses are reported only if the potential confounders substantially changed the intervention effect. Because the ITT and PP analyses yielded the same findings, only the ITT analyses are reported.

### Primary ^31^P-MRS outcomes

**Fig. 3:**
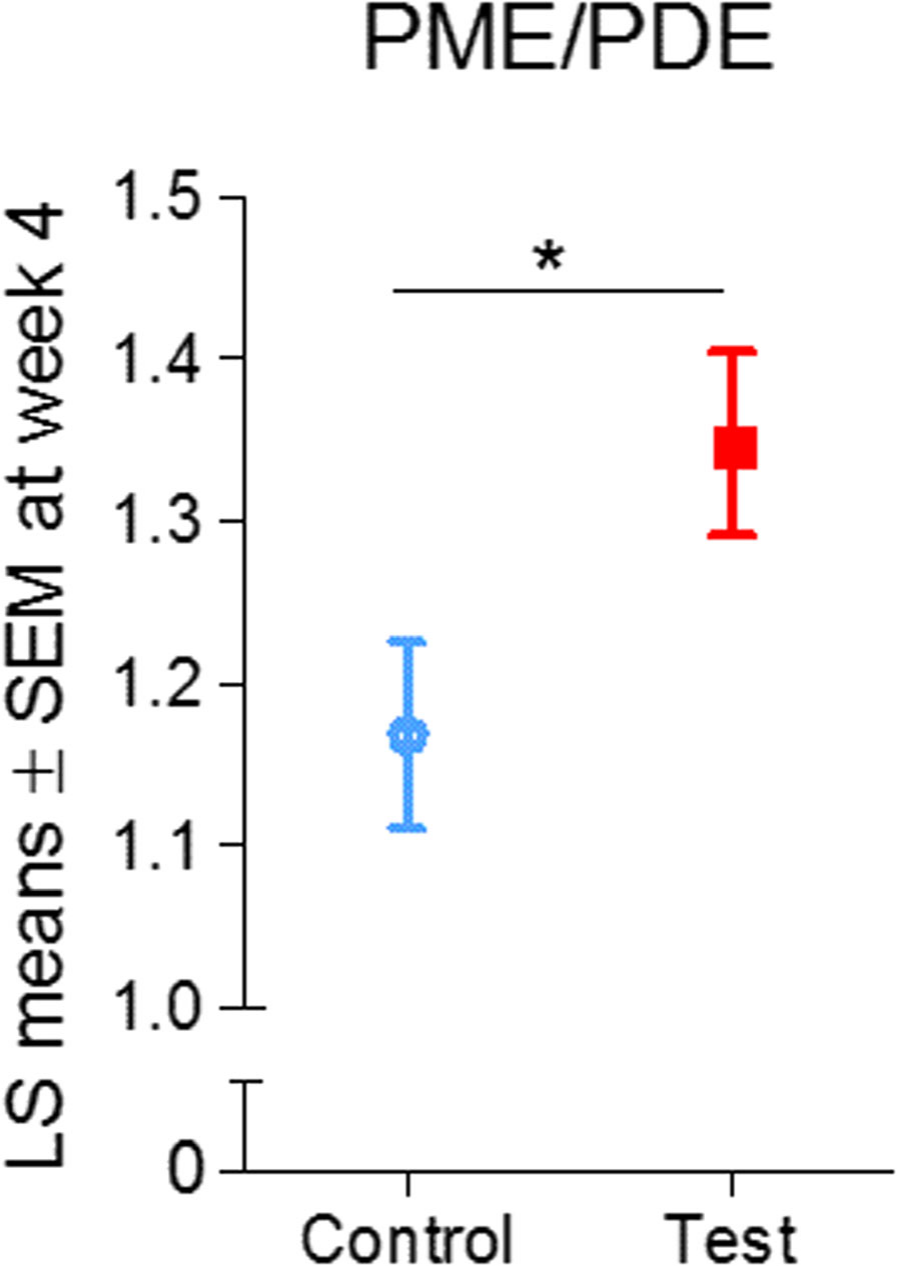
LS means ± SEM of PME/PDE at week 4 in the test and control groups. * *p* < 0.05. LS, Least squares; PME, Phosphomonoesters; PDE, Phosphodiesters

A significant intervention effect showing higher PME/PDE at week 4 in the test group than in the control group was found using the primary statistical analysis approach (i.e., predefined multilevel model) (least squares [LS] mean ± SEM test: 1.35 ± 0.06, control: 1.17 ± 0.06, *p* = 0.005) (Fig. 3). Visual inspection of the data and the results from the supportive model indicated that PME/PDE increased over time in the test group and decreased over time in the control group (*see* Additional file 1: Table S1, model 2).

No differences between groups were observed regarding the levels of PME LS mean ± SEM at week 4 (test: 12.05 ± 0.19, control: 11.93 ± 0.19, *p* = 0.628) or regarding levels of PDE (LS mean ± SEM at week 4, test: 9.79 ± 0.23, control: 9.89 ± 0.22, *p* = 0.702). For the results from the supportive models, *see* Additional file 1: Table S1.

### Other ^31^P-MRS outcomes

**Fig. 4:**
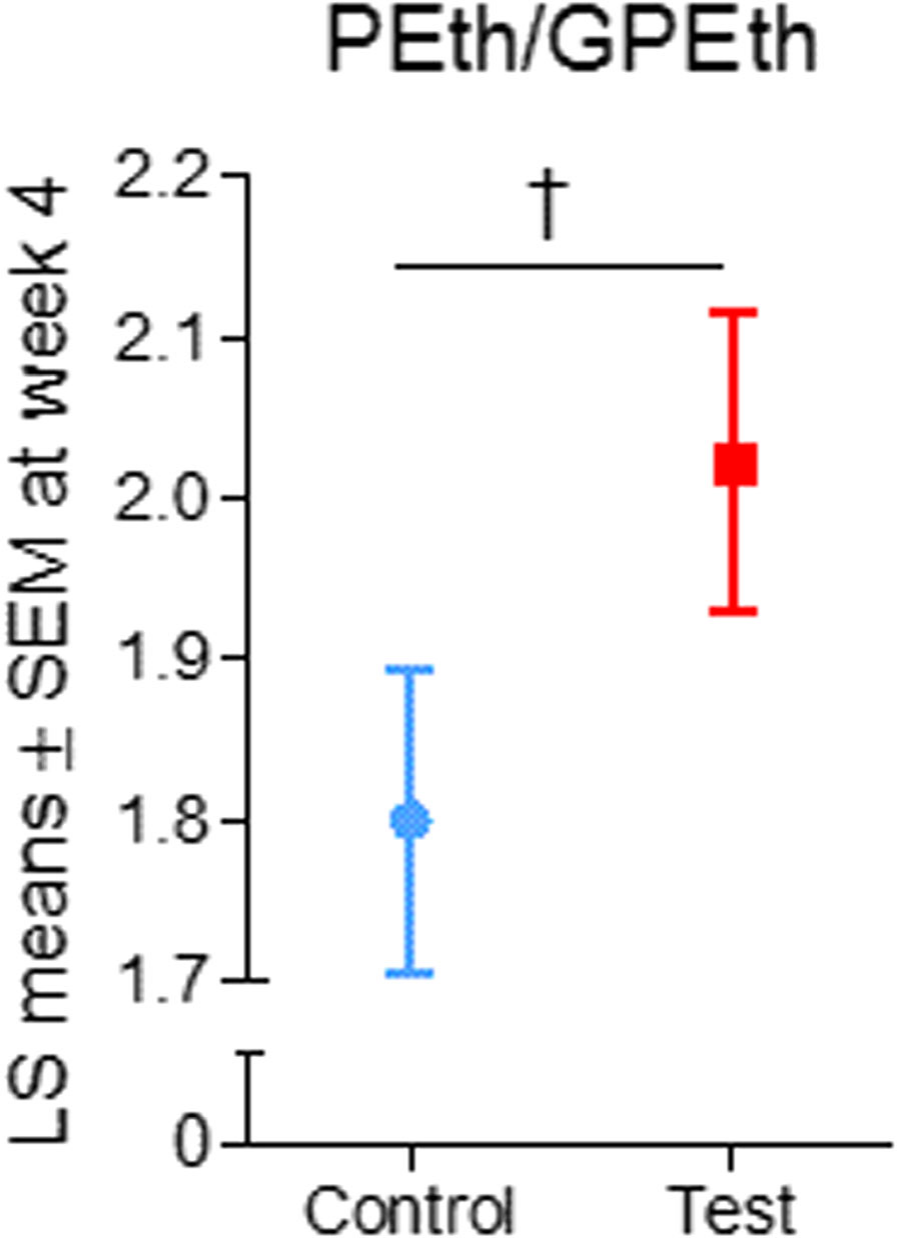
LS means ± SEM of PEth/GPEth at week 4 in the test and control groups. ^†^ *p* < 0.10. LS, Least squares; PEth, Phosphoethanolamine; GPEth, Glycerophosphoethanolamine

The PEth/GPEth ratio was higher in the test group than in the control group (LS mean ± SEM test: 2.02 ± 0.09, control: 1.80 ± 0.09, LS mean difference [95% CI] 0.22 [−0.04 to 0.48], *p* = 0.091) (Fig. 4). There were no significant differences between groups regarding the PCh/GPCh ratio; levels of PEth, PCh, GPEth, GPCh, PCr, Pi, ATP, or nicotinamide adenine dinucleotide; or pH (all *p* > 0.05).

### ^1^H-MRS outcomes

**Fig. 5:**
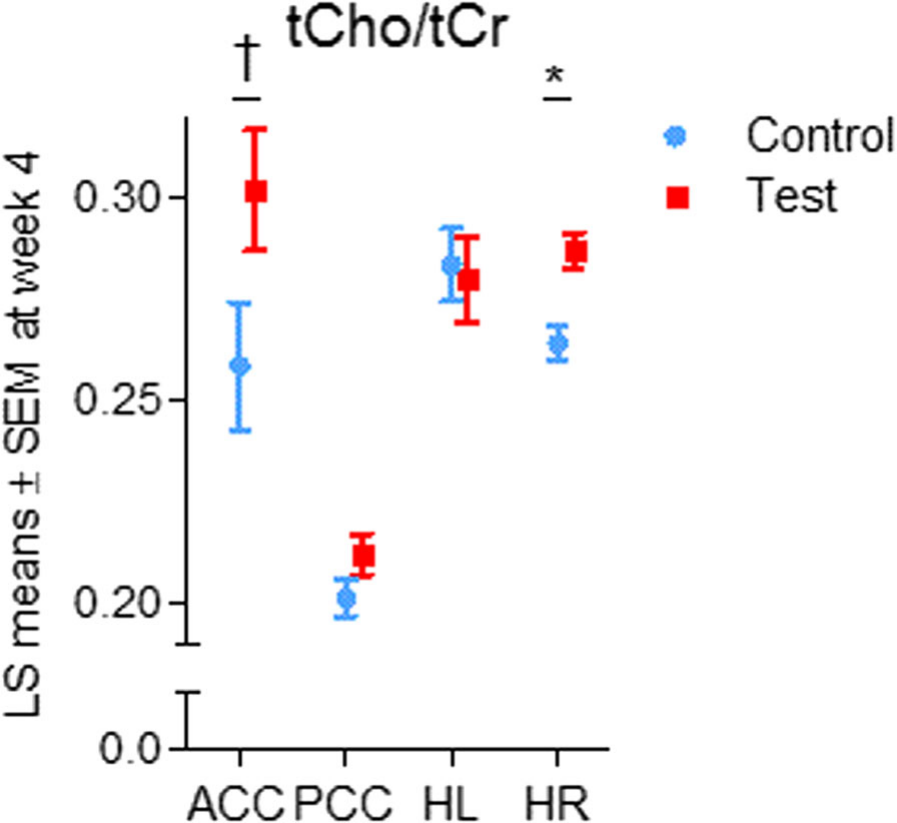
LS means ± SEM of tCho/tCr at week 4 in the test and control groups for four brain regions. * *p* < 0.05, ^†^ *p* < 0.10. LS, Least squares; tCho, Total choline; tCr, Total creatine; ACC, Anterior cingulate cortex; PCC, Posterior cingulate cortex; HR, Right hippocampus; HL, Left hippocampus

Using the primary statistical analysis approach, a significant interaction with brain region was found for tCho/tCr, leading to analyses per brain region. These showed that tCho/tCr at week 4 was higher in the test group than in the control group in the ACC (LS mean ± SEM test: 0.302 ± 0.015, control: 0.258 ± 0.016, *p* = 0.068) and in the HR (LS mean ± SEM test: 0.287 ± 0.004, control: 0.264 ± 0.004, *p* = 0.003), but not in the HL and PCC (both *p* > 0.05) (Fig. 5). For the results from the supportive models, *see* Additional file 1: Table S2.

For absolute levels of tCho, a significant intervention effect indicated that levels of tCho were higher at week 4 in the test group than in the control group (LS mean ± SEM test: 1.94 ± 0.05, control: 1.83 ± 0.05, *p* = 0.018; LS mean difference [95% CI] 0.11 [0.02 to 0.20], *p* = 0.018). There were no significant differences between groups regarding relative (i.e., relative to tCr) or absolute (i.e., water-referenced) levels of NAA or mI on the ratio NAA/mI or regarding absolute levels of tCre (all *p* > 0.05).

## Discussion

In this study, we investigated whether the medical food Souvenaid, containing nutritional precursors and cofactors for phospholipid membrane formation (i.e., FC), influenced brain phospholipid metabolism in mild AD. The observed effects indicate that this specific multinutrient combination not only raises circulating levels of phospholipid precursors after 4 weeks but also affects the balance between brain metabolites of phospholipid formation and breakdown in patients with mild AD. In addition, levels of tCho were higher after the intervention in comparison with the control product, whereas metabolic measures of neural integrity, gliosis, and energy metabolism were not significantly affected.

There is strong ex vivo evidence that in the AD brain, phospholipid content is decreased [3], phospholipid membrane composition is altered [3, 32], and phospholipid anabolic and catabolic processes are disturbed [7, 33], and a tight link of these changes with synaptic loss and synaptic dysfunction is presumed [32, 34]. The dependence of the unsaturated, low-affinity enzymes in the phospholipid synthesis pathway on substrate availability offers the opportunity to support this process by providing those substrates that are rate-limiting. Short-term supplementation with this specific multinutrient combination increases plasma and erythrocyte levels of nutritional precursors and cofactors for phospholipid membrane formation in patients with mild AD, which confirms and extends previous studies [27], and thus may alleviate preexisting nutritional deficiencies [14]. Because several mechanisms have been described that move key nutrients across the blood-brain barrier [9], the nutrients in the present investigation, or their metabolites, are expected to reach the brain. Accordingly, we observed a significantly increased PME/PDE ratio, which is considered to reflect the ratio of phospholipid anabolites over catabolites [22, 35], across four brain regions with 4 weeks of daily use of this multinutrient combination in mild AD. This indicates that the nutrients exert their effect on the brain’s phospholipid metabolism, in line with the hypothesized mode of action of this multinutrient combination. The observed changes in phospholipid metabolism may promote neuronal membrane formation and may stimulate dendritic spine formation, as was shown previously in animal studies [8, 9, 11]. The remodeling of phospholipid membranes in the brain will probably continue with prolonged intake, and thus continuous intake is needed for long-term effects on synapse formation. This may underlie the effects that this intervention has on memory in patients with mild AD [19, 20]. Future research is needed to confirm this hypothesis, as well as to investigate the actual impact of the changes in phospholipid metabolism on changes in synaptic function, functional connectivity and brain structural volumes in AD pathology, or on AD progression. Imaging techniques such as ^18^F-fluorodeoxyglucose positron emission tomography and structural MRI are currently used in ongoing randomized controlled trials to further explore whether this multinutrient combination affects synaptic function and structural brain volumes [36].

It was observed that the significantly increased PME/PDE ratio was driven by both an increased PME/PDE ratio in the active group and a decreased PME/PDE ratio in the control group. The decreased PME/PDE ratio in the control group was not expected over the course of 4 weeks of follow-up. The clinical significance of this decrease with respect to disease progression is currently unclear.

Because the PME/PDE ratio changed significantly but individual and total phosphomonoesters (PEth, PCh, and PME) and phosphodiesters (GPEth, GPCh, and PDE) did not, it cannot be established whether phospholipid formation was increased, breakdown was decreased, or both. In addition, the literature is inconclusive on in vivo levels of these metabolites in AD because increased, decreased, and unaltered levels of PME and PDE have been observed in comparison with cognitively normal controls [37–42]. However, the effect of this multinutrient combination on brain phospholipid metabolism appears to be driven mainly by the ethanolamine pathway because PEth/GPEth, but not PCh/GPCh, showed a trend toward an increase. This is in line with other studies that show that oral phospholipid precursors (pyrimidines and choline) in healthy populations [25, 26, 43] predominantly affect the ethanolamine pathway. However, in the present study, both relative and absolute tCho levels were higher at follow-up in the group receiving the multinutrient intervention than in the control group, indicating an effect on phosphocholine metabolism as well. The tCho signal reflects the choline-containing compounds PCh, GPCh, and free choline, and while both PCh and GPCh were measured separately with ^31^P-MRS, no increase was detected in either one. This discrepancy may have arisen because the measured ^1^H-MRS and ^31^P-MRS brain volumes differed, but supportive statistical models could not confirm the significant alterations of tCho after a 4-week intervention, warranting cautious interpretation. The analyses did reveal that changes in tCho may not be anatomically uniform, because the most prominent effects were observed in the ACC and HR. Because choline levels also increase in plasma, it is important to rule out that the measured increase in brain choline does not just reflect the vascular component of the measured volume. Using the available data, it was estimated that the change in plasma levels would constitute only about 0.2% of the change observed in brain choline, even when assuming that all plasma choline is MR-visible and estimating the vascular contribution at a liberal 5%.

Reduced levels of NAA and increased levels of mI have been shown consistently in AD [44], and it might have been expected that this multinutrient combination would decelerate the changes in these measures of neural integrity and gliosis. Although uridine and DHA were found to stimulate neurite outgrowth [45, 46], and DHA is known to have anti-inflammatory effects in addition to its role in the phospholipid synthesis pathway [47], no group differences after 4 weeks of intervention were observed regarding relative or absolute levels of NAA or mI, nor were differences observed regarding the NAA/mI ratio. However, the normal rate of change over 4 weeks’ time may be limited, such that a deceleration effect of the intervention is not yet discernible. Although 4 weeks of intervention did affect phospholipid metabolism, this period may have been too short to observe an effect on metabolic measures of neural integrity and gliosis. Alternatively, it may be that the damage that these metabolites reflect is already irreversible at this stage of the disease.

The findings of this study may be limited by the modest sample size. Although there was sufficient power to detect an effect on the PME/PDE ratio, one of the main outcome measures, it was not possible to ascribe the increase in this ratio to increased PME or decreased PDE. Moreover, MR spectra were not always available or of sufficient quality from all investigated brain regions. Finally, a longer intervention duration may have led to more robust findings and more pronounced secondary outcomes.

## Conclusions

This exploratory, double-blind, randomized controlled study shows that the medical food Souvenaid affects phospholipid metabolism across multiple brain regions in mild AD after only 4 weeks. This could lead to increased neuronal membrane formation, which would support the hypothesized mode of action of this multinutrient intervention. Larger and longer randomized controlled trials are needed to determine long-term effects on phospholipid formation, synaptic function, and cognition in persons with and at risk for AD.

## Additional file

Additional file 1: Supplement to ‘A. Rijpma, M. van der Graaf, M.M. Lansbergen, O. Meulenbroek, A. Cetinyurek-Yavuz, J.W. Sijben, A. Heerschap, and M.G.M. Olde Rikkert, The medical food Souvenaid affects brain phospholipid metabolism in mild Alzheimer’s disease: results from a randomized controlled trial’. (PDF 614 kb)

## Abbreviations

^1^H/^31^P-MRS: Proton/phosphorus magnetic resonance spectroscopy
ACC: Anterior cingulate cortex
AD: Alzheimer’s disease
AE: Adverse event
ANCOVA: Analysis of covariance
ATP: Adenosine triphosphate
BMI: Body mass index
Cr: Creatine
CRLB: Cramér-Rao lower bound
CSF: Cerebrospinal fluid
DHA: Docosahexaenoic acid
DPA: Docosapentaenoic acid
EPA: Eicosapentaenoic acid
FC: Fortasyn Connect
FOV: Field of view
GDS: Geriatric Depression Scale
GM: Gray matter
GPCh: Glycerophosphocholine
GPEth: Glycerophosphoethanolamine
HL: Left hippocampus
HR: Right hippocampus
ITT: Intention to treat
LC-PUFA: Long-chain polyunsaturated fatty acid
LS: Least squares
mI: *Myo*-inositol
MMSE: Mini Mental State Examination
MR: Magnetic resonance
MRI: Magnetic resonance imaging
MRS: Magnetic resonance spectroscopy
MRSI: 3D magnetic resonance spectroscopic imaging
NAA: *N*-acetylaspartate
PC: Phosphatidylcholine
PCC: Posterior cingulate cortex
PCh: Phosphocholine
PCr: Phosphocreatine
PDE: Phosphodiester
PE: Phosphatidylethanolamine
PEth: Phosphoethanolamine
Pi: Inorganic phosphate
PME: Phosphomonoester
PP: Per protocol
PRESS: Point resolved spectroscopy
ROI: Region of interest
RSC: Retrosplenial cortex
sLASER: Semilocalization by adiabatic selective refocusing
TA: Acquisition time
tCho: Total choline
tCr: Total creatine
TE: Echo time
TR: Repetition time
UMP: Uridine monophosphate
WM: White matter
