# Supplementary material for: The medical food Souvenaid affects brain phospholipid metabolism in mild Alzheimer’s disease: results from a randomized controlled trial
Source: Alzheimers Res Ther. 2017 Jul 26;9:51. doi: 10.1186/s13195-017-0286-2 (PMC5530581; doi:10.1186/s13195-017-0286-2)
Supplement: Additional file 1: — Supplement to ‘A. Rijpma, M. van der Graaf, M.M. Lansbergen, O. Meulenbroek, A. Cetinyurek-Yavuz, J.W. Sijben, A. Heerschap, and M.G.M. Olde Rikkert, The medical food Souvenaid affects brain phospholipid metabolism in mild Alzheimer’s disease: results from a randomized controlled trial’. (PDF 614 kb) [file 13195_2017_286_MOESM1_ESM.pdf]

Additional file 1

Supplement to: A. Rijpma, M. van der Graaf, M.M. Lansbergen, O. Meulenbroek, A. Cetinyurek-Yavuz, J.W. Sijben, A. Heerschap, and M.G.M. Olde Rikkert, The medical food Souvenaid affects brain phospholipid metabolism in mild Alzheimer's disease: results from a randomized controlled trial.

Results

Primary ^{31}P-MRS outcomes

Using the multilevel model with imputation, the intervention effect on PME/PDE was not significant (p=0.295) (Table A1). However, influential diagnostics revealed an influential subject that severely affected the results of the model. Excluding this subject from the multilevel model with imputation, resulted in a similar effect of the intervention on PME/PDE at week 4 as observed using the primary analysis approach (LS mean±SEM test: 1.29±0.06; control: 1.16±0.05, p=0.061, Table A1 Model A). Results from the other two supportive models aligned with the primary analysis approach (for details, see Table A1, Model 2-3). The supportive mixed model with time resulted in a significant intervention group by time interaction, which expresses the intervention effect (for details, see Table A1, Model 2).

No differences between groups were observed regarding the levels of PME (all models p>0.05) or regarding levels of PDE (all models p>0.05).

^{1}H-MRS outcomes

Using the multilevel model with imputed baseline, an overall significant intervention effect was found on relative tCho levels indicating that tCho/tCr at week 4 was higher in the test group than in the control group (Table S2; LS mean±SEM test: 0.264±0.004; control: 0.252±0.004, p=0.019).

In the supportive mixed model with time of tCho/tCr, the p-value of the three-way interaction between intervention, brain region and time was below the pre-specified threshold (p<0.10), leading to per brain region analyses. The intervention had no significant interaction with time at any of the brain regions (Table A2; all p>0.05).

Table S1. Results from statistical analysis for PME/PDE; ITT population.

	N Test / N Control	Mean estimated value at week 4		Mean estimated group difference at week 4 (95% CI)	p-value
		Test	Control		
**PME/PDE**					
Primary analysis approach					
	14/15	1.35	1.17	0.18 (0.06,0.30)	0.005
Supportive models					
- Model 1	15/16	1.25	1.17	0.08 (-0.07,0.23)	0.295
- Model 1b	14/16	1.29	1.16	0.14 (-0.01,0.28)	0.061
- Model 2	16/17	0.07*	-0.09*	0.16 (0.02,0.31)†	0.024#
- Model 3	14/15	1.44	1.27	0.17 (0.02,0.33)	0.030

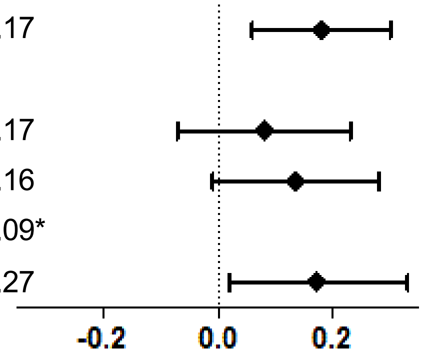

Note. Model 1: multilevel model with imputation; Model 1b: Model 1 excluding influential subject; Model 2: mixed model with time; ; Model 3: multilevel model with most complete brain region (i.e. retrosplenial cortex). Forest plots reflect mean estimated group differences at week 4 with 95% CI. PME, phosphomonoesters; PDE, phosphodiesters; PEth, phosphoethanolamine; GPEth, glycerophosphoethanolamine; ITT, intention to treat; CI, confidence interval; BL, baseline; na, not applicable. *Mean estimated change from baseline, †mean estimated group difference in change from baseline, #p-value for interaction between intervention and time (in days).

Table S2. Results from statistical analysis for tCho; ITT population.

	N Test / N Control	Mean estimated value at week 4			Mean estimated group difference at week 4 (95% CI)	p-value
		Test	Control			
**Relative tCho (tCho/tCr)**						
Primary analysis approach						
- ACC	10/8	0.30	0.26	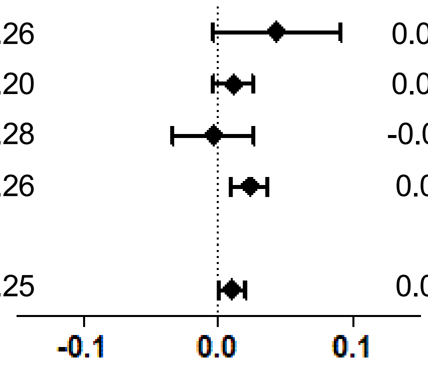	0.04 (-0.00,0.09)	0.068
- PCC[1]	12/14	0.21	0.20		0.01 (-0.00,0.02)	0.139
- HL	9/11	0.28	0.28		-0.00 (-0.03,0.03)	0.794
- HR	9/10	0.29	0.26		0.02 (0.01,0.04)	0.003
Supportive models						
- Model 1	14/16	0.26	0.25		0.01 (0.00,0.02)	0.019
- Model 2						
- ACC	14/11	-0.004*	-0.023*		0.019 (-0.047,0.085)[†]	0.578[#]
- PCC	14/16	0.006*	-0.004*		0.010 (-0.005,0.024)[†]	0.197[#]
- HL	12/15	-0.015*	-0.000*		-0.015 (-0.041,0.012)[†]	0.290[#]
- HR	12/14	0.003*	0.005*		-0.002 (-0.028,0.023)[†]	0.869[#]

Note. Model 1: multilevel model with imputation; Model 2: mixed model with time; [1]Supportive model 3: multilevel model with most complete brain region (posterior cingulate cortex); Forest plots reflect mean estimated group differences at week 4 with 95% CI. ITT, intention to treat; CI, confidence interval; BL, baseline; na, not applicable. *Mean estimated change from baseline, [†]mean estimated group difference in change from baseline, [#]p-value for interaction between intervention and time (in days).

Table S3. Means and standard deviations of the mean of the blood outcome parameters at baseline.

	**Test n=16**	**Control n=17**
Fatty acids in the erythrocyte membrane		
- DHA (%)	2.9 (0.8)	2.9 (0.9)
- EPA (%)	0.8 (0.3)	0.8 (0.3)
- DPA (%)	1.7 (0.4)	1.6 (0.3)
- LC-PUFA (=DHA+EPA+DPA) (%)	5.4 (1.2)	5.3 (1.2)
Fatty acids in blood plasma		
- DHA (%)	1.6 (0.5)	1.6 (0.5)
- EPA (%)	0.8 (0.3)	0.8 (0.4)
- DPA (%)	0.5 (0.1)	0.5 (0.1)
- LC-PUFA (=DHA+EPA+DPA) (%)	3.0 (0.8)	2.9 (0.8)
Choline levels in blood plasma (uM)	8.4 (1.8)	9.1 (2.2)
Vitamin E levels in blood plasma (uM)	39.5 (7.0)	39.8 (9.2)
Homocysteine levels in blood plasma (uM)	11.5 (2.9)	11.6 (3.2)
Uridine levels in blood plasma (uM)	3.7 (1.4)	3.0 (1.3)

Note. Data presented are as mean (SD). DHA, docosahexaenoic acid; EPA, eicosapentaenoic acid; DPA; docosapentaenoic acid; LC-PUFA, long-chain polyunsaturated fatty acids.

Table S4 - Means and standard deviations of the mean at baseline for the main ^{31}P-MRS and main ^{1}H-MRS outcome parameters.

^{31}P-MRS	ACC		RSC		HR		HL	
	Test n=16	Control n=17	Test n=16	Control n=17	Test n=16	Control n=17	Test n=16	Control n=17
PME/PDE	1.05 (0.21) [10]	1.21 (0.22) [12]	1.27 (0.22) [15]	1.42 (0.24) [16]	1.28 (0.30) [14]	1.16 (0.21) [12]	1.10 (0.28) [12]	1.23 (0.18) [13]
PME	12.47 (1.80) [15]	12.39 (1.57) [15]	11.19 (1.45) [15]	12.05 (1.53) [17]	12.32 (1.62) [15]	11.49 (0.59) [13]	11.97 (1.40) [14]	12.67 (2.17) [14]
PDE	11.92 (2.21) [10]	10.33 (1.79) [13]	9.03 (0.91) [16]	8.52 (1.24) [16]	9.90 (1.95) [14]	10.58 (1.82) [14]	10.68 (2.52) [14]	10.56 (1.40) [14]
^{1}H-MRS	ACC		PCC		HR		HL	
	Test n=16	Control n=17	Test n=16	Control n=17	Test n=16	Control n=17	Test n=16	Control n=17
NAA/tCr	1.04 (0.23) [14]	1.11 (0.14) [12]	1.14 (0.08) [12]	1.16 (0.10) [16]	1.08 (0.15) [11]	1.02 (0.13) [12]	1.09 (0.20) [11]	0.97 (0.10) [12]
mI/tCr	0.72 (0.19) [14]	0.81 (0.17) [12]	0.80 (0.10) [12]	0.89 (0.19) [16]	1.23 (0.11) [11]	1.36 (0.22) [12]	1.22 (0.20) [11]	1.36 (0.16) [12]
tCho/tCr	0.30 (0.09) [12]	0.28 (0.05) [10]	0.21 (0.02) [12]	0.20 (0.02) [15]	0.29 (0.05) [10]	0.25 (0.04) [12]	0.31 (0.07) [10]	0.28 (0.03) [12]
NAA/mI	1.53 (0.47) [14]	1.44 (0.41) [12]	1.45 (0.24) [12]	1.36 (0.29) [16]	0.89 (0.16) [11]	0.78 (0.19) [12]	0.90 (0.14) [11]	0.72 (0.12) [12]

Note. Data presented are as mean (SD) [N]. PME, phosphomonoesters; PDE, phosphodiesters; NAA, N-acetylaspartate; mI, myo-inositol and glycine; tCr, total creatine; tCho, total choline; ACC, anterior cingulate cortex; RSC, retrosplenial cortex; HL and HR, left and right hippocampus; PCC, posterior cingulate cortex.
